# Trigeminal neuropathy: Two case reports of gasserian ganglion stimulation

**DOI:** 10.1002/brb3.2379

**Published:** 2021-10-17

**Authors:** Yannick Logghe, Iris Smet, Ali Jerjir, Peter Verelst, Marieke Devos, Jean‐Pierre Van Buyten

**Affiliations:** ^1^ Multidisciplinary Pain Center AZ Nikolaas Sint‐Niklaas Belgium; ^2^ Department of Anesthesiology AZ Nikolaas Sint‐Niklaas Belgium

**Keywords:** gasserian ganglionpain, neuromodulation, pain, trigeminal neuropathypain

## Abstract

This report describes the successful treatment of two patients with trigeminal neuropathy by using gasserian ganglion stimulation.

**Case reports**: The first case report deals with a 53‐year‐old woman suffering from right‐sided facial pain after a gamma knife lesion for schwannoma of the right inner ear. For 9 years, several interventions with the aim of relieving the pain were unsuccessful; in fact, they had aggravated the symptoms. A trial with a neurostimulator at the level of the Gasser ganglion had an immediately positive effect on her score for facial pain, which decreased from 7.3 to 0 on a visual analog scale, assessed during a period of 2 months. Additionally, the patient had weaned off all her medication by the end of the period. The second case report describes a 64‐year‐old man suffering from trigeminal neuropathy, which mainly manifested itself as an itch. For a period of 15 years, neither medication nor several interventions were effective. A trial with an electrode at the level of the Gasser ganglion reduced his pain score from 7.0 to 1.5 on a visual analog scale, assessed during a period of three months. His medication could be limited to pregabalin 150 mg bidaily. In contrast, prior to the implantation, his oral medication consisted of pregabalin 75 mg up to five times a day.

**Conclusion**: These case reports show that stimulation of the gasserian ganglion is a successful, minimally invasive, and non‐destructive treatment in refractory trigeminal neuropathy and should be considered earlier in the treatment algorithm of trigeminal neuropathy.

## Graphical abstract

The manuscript reports two case reports on the use of neuromodulation in trigeminal neuropathy. Both patients had an extensive history of interventions that had no effect on their symptoms, which in fact aggravated their complaints. Our case reports show the successful result of stimulation of the gasserian ganglion in trigeminal neuropathy.

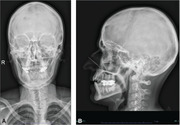

## 1 INTRODUCTION

Trigeminal neuropathy is defined as the facial pain in the distribution of one or more branches of the trigeminal nerve. The pain is usually continuous and described as burning or squeezing. It is often accompanied by mechanical allodynia and cold hyperalgesia. Facial pain has been classified by Burchiel based on symptoms and history (Burchiel, 2003). A more recent classification is published by the International Headache Society. Both classifications are shown in Table 1. Following the classification of the International Headache Society, trigeminal neuropathy usually has a cause, which can be a traumatic event, such as a mechanical, chemical, thermal lesion or radiation. It can be secondary to a postherpetic infection (Olesen, 2018). Trigeminal neuropathy should not be confused with trigeminal neuralgia. Trigeminal neuralgia is divided into three subclasses: classical trigeminal neuralgia, caused by a neurovascular compression of the trigeminal nerve solely; secondary trigeminal neuralgia, caused by an underlying disease such as an atrioventricular malformation, skull‐base bone deformity or multiple sclerosis; idiopathic trigeminal neuralgia with neither electrophysiological tests nor magnetic resonance imaging showing significant abnormalities. Trigeminal neuralgia is described as a paroxysmal pain, abrupt in onset and termination. It is perceived as an electric shock‐like pain.

**TABLE 1:** Comparison: Burchiel's classification and the classification of the International Headache Society (IHS)

Burchiel's classification	History/pattern	Causes	IHS classification	Causes
**Spontaneous onset**			**Trigeminal neuralgia**	
TN type I	>50% paroxysmal pain	Neurovascular compression of trigeminal nerve or unknown	Classical trigeminal neuralgia, purely paroxysmal	Neurovascular compression exclusively
TN type II	<50% paroxysmal pain	Neurovascular compression of trigeminal nerve or unknown	Classical trigeminal neuralgia with concomitant continuous pain	Classical trigeminal neuralgia with persistent background facial pain
Symptomatic TN	TN due to multiple sclerosis, tumors, etc.	Demyelination	Secondary trigeminal neuralgia	Due to multiple sclerosis, tumor, AV‐malformation, etc.
			Idiopathic trigeminal neuralgia	Trigeminal neuralgia with neither electrophysiological tests nor MRI abnormalities
**Atypical facial pain**			**Painful trigeminal neuropathy**	
*Peripheral trigeminal injury* TNP	Incidental nonintentional injury	ENT/oral surgery, facial trauma, stroke, etc.	Painful post‐traumatic trigeminal neuropathy	Mechanical, chemical, thermal, or caused by radiation. Post neuroablative procedures for trigeminal neuralgia
Trigeminal deafferentation pain	Trigeminal injury from peripheral ablation	RF rhizotomy, glycerol rhizolysis, GKR balloon compression, etc.		
			PTN attributed to other disorders	Secondary to multiple sclerosis, space‐occupying lesion or systemic disease, with only the clinical characteristics (quality of spontaneous pain, evoked pain and presence of sensory deficits)
**Postinfection**				
Postherpetic neuralgia	Herpes zoster outbreak	Shingles involving trigeminal distribution	PTN attributed to herpes zoster	Unilateral facial pain of less than 3 months’ duration caused by and associated with other symptoms and/or clinical signs of acute herpes zoster
			Trigeminal post‐herpetic neuralgia	Unilateral facial pain persisting or recurring for at least 3 months
			Idiopathic PTN	Unknown etiology with clinically evident positive (hyperalgesia, allodynia) and/or negative (hypaesthesia, hypalgesia) signs of trigeminal nerve dysfunction

*Abbreviations*: ENT, ear, nose and throat; GKR, gamma‐knife radio surgery; RF, radio frequency; TN, trigeminal neuralgia; TNP, trigeminal neuropathic pain.

Our first case study concerns a patient suffering from trigeminal neuropathy caused by a gamma knife lesion, which manifests itself as a painful, tingling, and cold sensation. In our second case study, trigeminal neuropathy manifests itself as an itching sensation. Itching is defined as an uncomfortable sensation causing a desire to scratch. Itching becomes chronic if it persists after 6 weeks (Yosipovitch et al., 2018). Itch‐sensitive neurons can be divided into two subtypes: histaminergic neurons and non‐histaminergic neurons. Histaminergic neurons are activated in acute itching, and chronic itching is not induced by histamine (Yosipovitch et al., 2018). Chronic itching has well‐recognized similarities with neuropathic pain also known as neuropathic itching. The same neuromediators are found in chronic itching and chronic pain including substance P, opioids, nerve growth factor, neurotrophin 4, and proteases (Yosipovitch et al., 2007).

## 2 CURRENT TREATMENT STRATEGIES

Treatment of painful trigeminal neuropathy starts with tricyclic antidepressants (TCA) or anticonvulsants if TCA's are contra‐indicated. If therapy with TCA's is insufficient, gabapentin or pregabalin is tried. The next steps consist of further trials with combinations of TCA's and serotonin and noradrenalin reuptake inhibitors (SNRI's). Trigeminal neuralgia therapy usually starts with carbamazepine. In comparison with patients with trigeminal neuralgia, patients with a trigeminal neuropathy do not respond well to pharmacological therapy (Haviv et al., 2014). In case of a trigeminal neuropathy which manifests as an itch, no specific antipruritic drugs have been developed. Due to the similarities with chronic pain, antidepressants and anticonvulsants are also administered for inhibition of itching. Topical agents such as capsaicin, aspirin, and salicylates are used for more localized chronic itching. Oral cyclo‐oxygenase inhibitors do not ameliorate pruritus (Yosipovitch et al., 2007). Immunosuppressants, such as thalidomide and methotrexate, are used for dermatological diseases, but potentially have severe side effects with long‐term use (Yosipovitch et al., 2018).

Invasive procedures, such as microvascular decompression (MVD), radiofrequency ablations, and gamma knife procedures are proven not to be effective in patients with trigeminal neuropathy. Moreover, in 73% of the patients symptoms are worsening (Mehrkens & Steude, 2007; Sweet, 1988). Neuromodulation techniques such as motor cortex stimulation and deep brain stimulation targeting subcortical regions have possible severe complications such as seizures, deep electrode infection leading to sepsis, ventricular hemorrhage and is therefore not recommended (Antony et al., 2019).

Neuromodulation at the level of the gasserian ganglion has been used successfully in the treatment of neuropathic facial pain and is appropriate in patients with trigeminal neuropathy, i.e. a lesion of the trigeminal nerve, iatrogenic or from another cause (Mehrkens & Steude, 2007). Due to the overlap between chronic pain and chronic itching, neuromodulation could have a positive effect in these patients, as stimulation of glycinergic dorsal horn neurons alleviates pain perception (Foster et al., 2015).

This case report describes the use of neurostimulation of the gasserian ganglion in two patients with a trigeminal neuropathy for whom all previous therapies have failed.

## 3 FIRST CASE

**FIGURE 1:**
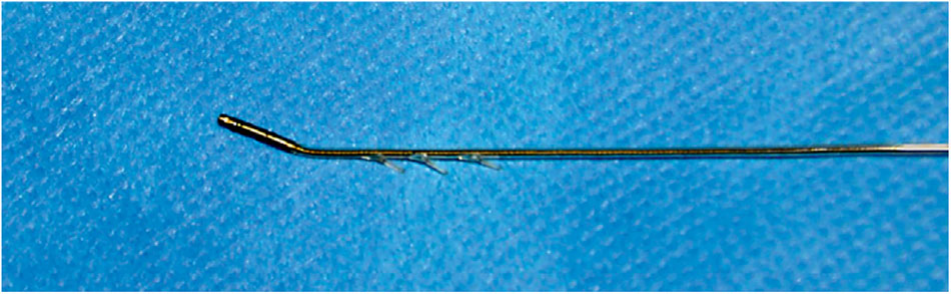
Custom‐made tripolar bent tined lead (Medtronic BRC/Van Buyten) (Van Buyten, 2015)

**FIGURE 2:**
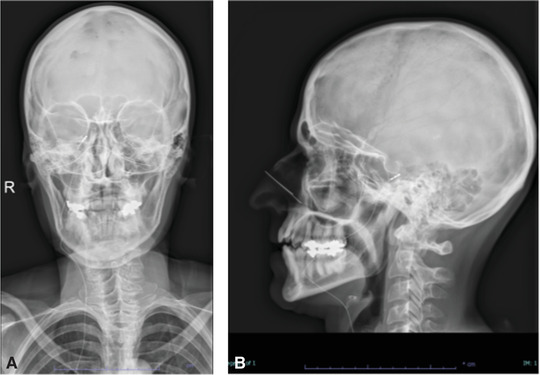
Skull‐base X‐ray of the first case (a) anteroposterior view, (b) lateral view. Visualization of the tripolar electrode placed through the foramen ovale. The electrode is tunneled subcutaneously along the neck to the right infraclavicular fossa

**FIGURE 3:**
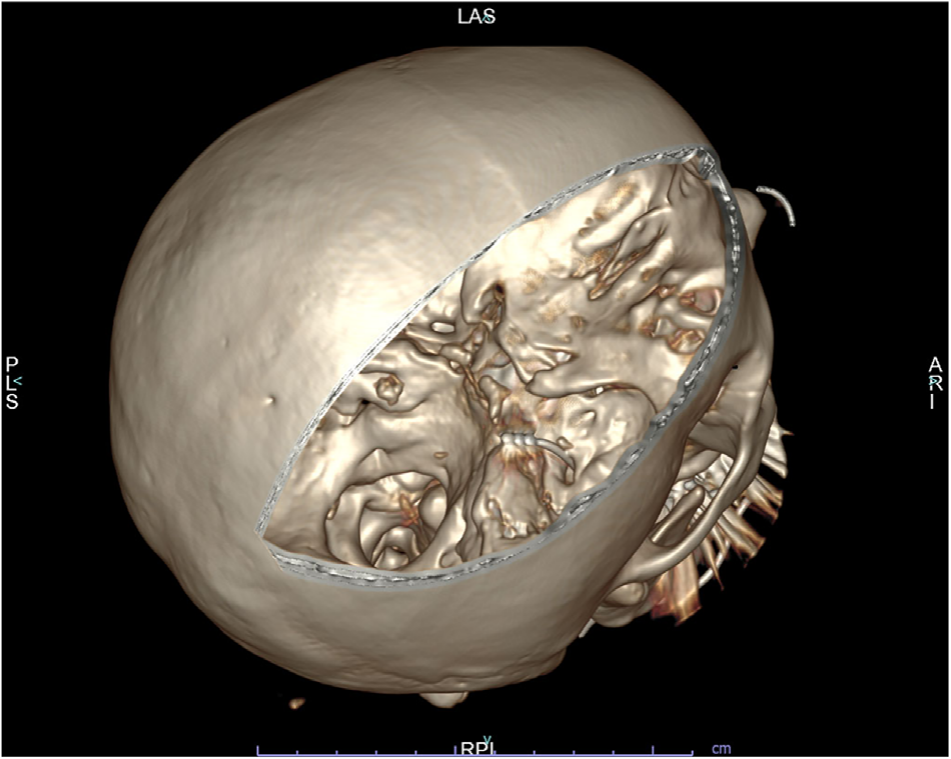
CT image reconstruction of the first case. Visualization of the tripolar electrode clearly bent over the gasserian ganglion

A 53‐year‐old woman suffered from a trigeminal neuropathy of all three trigeminal branches on the right side of the face. She rated her pain 7.3/10 on a visual analog scale (VAS) and reported pain 20 days a month with frequent intense attacks of 8–10 times a day. She described the pain as tingling and cold. The patient had an extensive medical history with several interventions to relieve the pain. She was known with a cluster headache since 2005, featuring only one attack a month. After a gamma knife lesion for schwannoma of the right inner ear, a trigeminal neuropathy was reported in 2012. Moreover, she started suffering from more frequent cluster headache attacks. Between 2012 and 2019, several procedures were performed aiming to treat the cluster headache attacks. These procedures included occipital nerve infiltrations, radiofrequency ablation of the gasserian ganglion and the sphenopalatine ganglion, physiotherapy, hypnosis, and acupuncture. None of them appeared to be effective. An occipital neurostimulator was advised, yet never implanted due to the absence of reimbursement. Instead, a gamma knife lesion of the sphenopalatine ganglion was executed to counter the attacks of cluster headache. However, the number of attacks increased to 8–10 times a day. The patient was very disabled because of a continuous trigeminal neuropathy in combination with the frequent cluster headache attacks. A final MVD resulted in only short‐term pain relief. At the beginning of 2021, a trial with a neurostimulator at the level of the gasserian ganglion was planned on the right side. Prior to the implant of the electrode, a 3D‐computed tomography (CT) scan of the head was performed and transferred to the neuronavigation machine (Van Buyten, 2015). During the operation, the patient was kept under general anesthesia in the supine position. Based on the Sweet technique (Sweet & Wepsic, 1974), a small incision was made 1–3 cm lateral to the right labial commissure of the mouth. The electromagnetic neuronavigation guidance consists of a 14‐Gauge modified Tuohy needle (MDT®) equipped with two magnetic coils, inserted into a thermocouple needle which is commonly used for radiofrequency procedures. The tip of the needle was tracked on his path to the foramen ovale through which a custom‐made tripolar bent, tined lead (custom‐made Medtronic/Van Buyten) was inserted (Figure 1) (Van Buyten et al., 2009). The tines attach to the soft tissue inferior to the foramen ovale and do not enter the foramen. Peroperative motor threshold was measured at 2 Hz. The needle was withdrawn under continuous fluoroscopy to assure the electrode remains in the correct position. The electrode was tunneled subcutaneously in a straight track over the submandibular region along the neck to the ipsilateral infraclavicular fossa. An incision for the electrode is made superficially to the scalenus lodge, lateral to the external jugular vein. At the infraclavicular area, the electrodes are connected to a temporary extension wire to start a trial. The initial frequency was set to 50 Hz and pulse width at 450 μs. Long battery life can be expected since low voltages are used for stimulation. A skull‐base X‐ray (Figure 2) and CT scan were obtained (Figure 3). The tripolar electrode is clearly bent over the gasserian ganglion. Two weeks after the start of the trial she rated her facial pain 2/10, reporting as a tingling sensation. Only a pinpoint stimulation was felt next to the nostril. The implantable pulse generator (IPG) was placed under general anesthesia. The previous infraclavicular incision was reopened and the temporary extension was removed. An IPG (Intellis®, Medtronic) was implanted in the infraclavicular fossa. Since the implantation, facial pain attacks have no longer occurred. Her quality of life has significantly improved and she is no longer disabled. Prior to the implantation, her oral medication consisted of pregabalin 100 mg in the morning and pregabalin 225 mg as well as amitriptyline 10 mg in the evening. Four weeks after the start of the trial, her pain medication has been significantly reduced to pregabalin 75 mg in the evening. The patient rated her patient global impression of change (PGIC) as very satisfied. One week after this last follow‐up, she consulted with an intraoral erosion of the electrode. Under local anesthesia, the electrode was successfully covered by marsupialization. Ten days after this procedure, she reported a pain score of 0/10 and did no longer take any medication.

## 4 SECOND CASE

**FIGURE 4:**
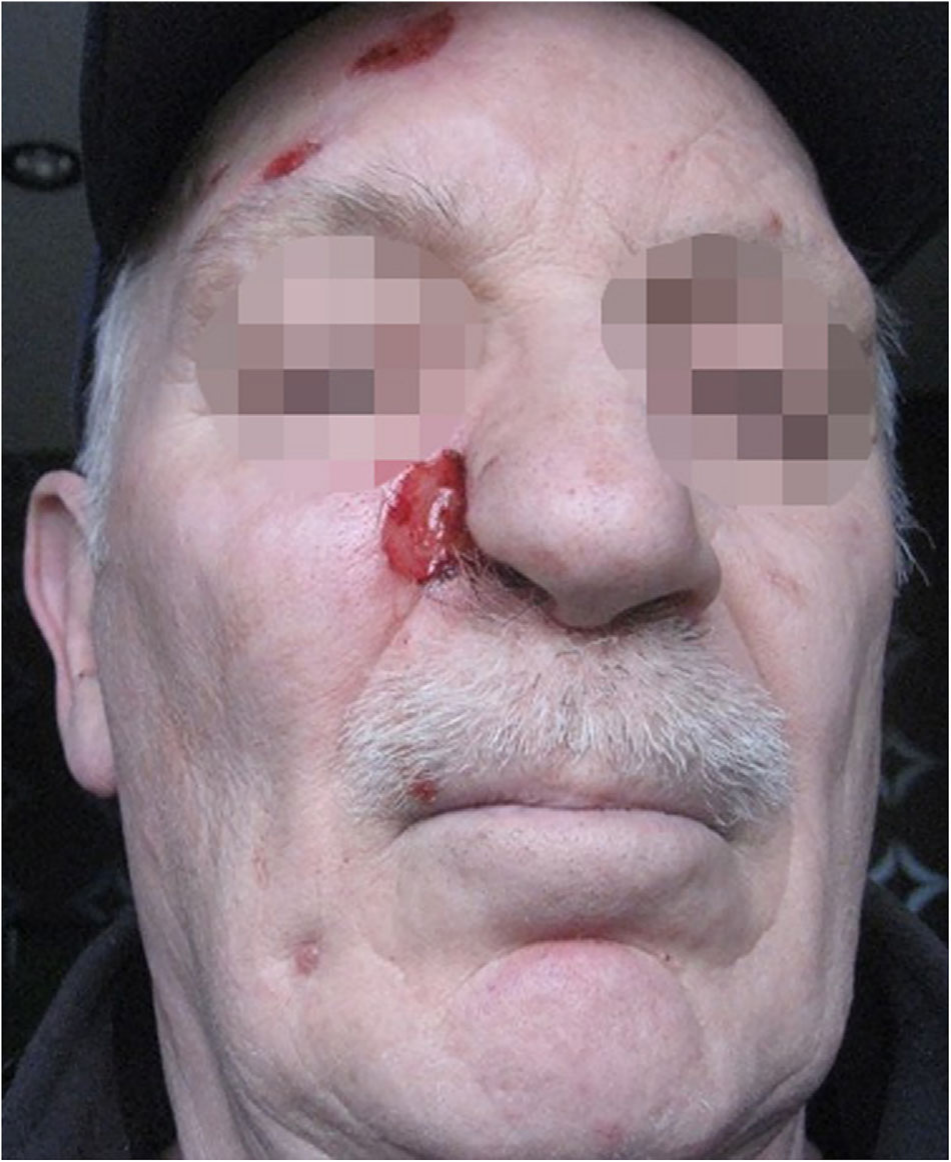
Second case. Ulcers on the right nostril (ala nasi) and above the right eyebrow caused by scratching as a result of neuropathic itching

A 64‐year‐old man suffered from trigeminal neuropathy, which mainly manifested as an itch near the right eyebrow and on the right nostril. This resulted in scratching during his sleep, causing ulcers on the right nostril (ala nasi) and the right eyebrow (Figure 4). Otherwise, he had no significant medical history. In the past, he only had an episode of atrial fibrillation, for which nebivolol and acetylsalicylic acid had been prescribed.

Since 1980, the patient had trigeminus neuralgia in the second branch of the trigeminal nerve. An alcoholization of the infraorbital nerve was performed, which reduced the complaints. Since he suffered from recurrent pain, an MVD was performed with favorable results lasting for 10 years. After 10 years the pain reappeared, but a second MVD did not result in the same pain relief. In 2004, the patient developed a typical trigeminal neuropathy that mainly manifested as an itch and unpleasant feeling on the right nostril (ala nasi) and above the right eye. At this time, his daily pain score on a VAS was 7.0/10. His quality of life deteriorated badly because of the severity of the itching and the impact on his daily life. Two radiofrequency ablations of the gasserian ganglion were not effective. In the following years’ the treatment focused on drug therapy. Neither gabapentin, pregabalin, carbamazepine nor lamotrigine were effective. A trial with neurostimulation at the level of the gasserian ganglion was planned. The same implantation technique as in the first case was used to implant a tripolar electrode at the level of the gasserian ganglion (Van Buyten et al., 2009). A trial stimulation was performed for 5 weeks. The stimulation reduced the itching and tingling. A battery (type Itrel 4®, Medtronic) was implanted at a second stage also in the infraclavicular fossa on the right and connected to the electrode. A skull‐based X‐ray was obtained.

Three months after the procedure, the patient's VAS score had dropped to 1.5/10. The patient rated his PGIC as very satisfied. He reported some minor tingling's during nighttime. His oral medication was adjusted to pregabalin 150 mg bidaily. In contrast, prior to the implantation, his oral medication consisted of pregabalin 75 mg up to five times a day. In the following months, the dose of oral medication was further reduced to pregabalin 75 mg in the morning and 150 mg in the evening; though, an attempt to reduce pregabalin even further was unsuccessful. This can be explained by the fact that the neurostimulator was accidentally turned off at the time of the second follow‐up at the pain center. Ten months after implantation, the VAS pain score remained 1.8/10 and the patient was satisfied. One and a half year after implantation, the patient was still on the same dose of pregabalin with a pain score of 2/10 and was satisfied with the therapy. Although the itchiness was mainly resolved, the scratching had become an unconscious habit over the years. Consequently, the scratch marks had not disappeared but improved. No adverse events were noted.

## 5 DISCUSSION

This report describes two cases of trigeminal neuropathy with an iatrogenic cause. Both patients show a similar, extensive history of interventions related to the pathology. When the neuropathy appeared, no clear distinction was made between trigeminal neuralgia and trigeminal neuropathy. Both patients were treated with radiofrequency ablations of the gasserian ganglion and/or sphenopalatine ganglion, which can be a good therapy in trigeminal neuralgia, but often aggravates symptoms in trigeminal neuropathy (Kustermans et al. 2017). Correctly diagnosing the trigeminal neuropathy should bring neuromodulation in the treatment algorithm earlier, taking into account that the technique is minimally invasive and non‐destructive. Besides, the study of Kustermans et al. (2017), including 17 patients, demonstrates that this technique gives at least 50% pain relief in 44% of patients on a long‐term basis. This percentage is higher compared to other surgical techniques (Kustermans et al. 2017).

The dorsal root ganglion is responsible for the transmission of signals from the peripheral to the central nervous system and is involved in the control over nociceptive signals, as described in the gate control theory of pain by Melzack and Wall (Foster et al., 2015). The dorsal root ganglion plays a significant role in the development of chronic pain. The gasserian ganglion can be considered as the dorsal root ganglion of the face, since it has the same role in the transmission of signals from the peripheral trigeminal branches to the central nervous system as the dorsal root ganglia do from the peripheral nervous system to the dorsal horns of the spinal cord at cervical, thoracic, lumbar and sacral levels. Stimulation of the ganglion makes it possible to selectively target the trigeminal branches. Studies have shown the successful analgetic effect of electrostimulation of the gasserian ganglion (Mehrkens & Steude, 2007; Kustermans et al. 2017). However, some complications, such as electrode dislocation (10–30%) and other mechanical defects (24%), have been reported. The use of peripheral nerve stimulation is not yet recommended for trigeminal facial pain by the international headache society (Deer et al., 2014). This recommendation is primarily based on a study issued by Taub et al. in 1997 (Taub et al., 1997). In our patients, we use a more advanced technique. Continuous adaptation of the implant technique has improved the results and decreased the number of adverse events. Recent improvements include the use of CT‐based electromagnetic neuronavigational guidance, in which the tip of the needle is tracked on its path to the foramen ovale (Van Buyten et al., 2009). A custom‐made tripolar bent electrode (custom‐made Medtronic/Van Buyten) is inserted through the foramen. The design of the electrode makes it possible to cover the gasserian ganglion (Figure 3). In addition, three tines are attached to fix the electrode near the entrance of the foramen ovale and avoid dislocation (Kustermans et al. 2017). The electrode is tunneled subcutaneously directly to the infraclavicular fossa and no longer preauricularly. This prevents the development of the previous complication of preauricular erosion. A trial stimulation is generally performed during 4 to 5 weeks, unless the patient chooses otherwise. If the stimulations give a good reduction of symptoms an IPG is implanted in the infraclavicular fossa or tunneled towards the abdominal wall depending on the patient's physiognomy or preference and connected to the electrode. The type of IPG depends on the patient's choice regarding energy consumption, shape, and price of rechargeable and non‐rechargeable batteries.

To date, there are no clear treatment algorithms for the treatment of trigeminal neuropathy. These cases prove that neurostimulation of the gasserian ganglion has a place in the algorithm. Considering the minimal invasiveness of the procedure, this therapy should be considered before neurodestructive procedures such as gamma knife treatments or more invasive neuromodulation therapies such as motor cortex stimulation. Our cases took 9 and 15 years between the onset of symptoms and the implantation of a neurostimulator. In spinal cord stimulation, the time between the onset of symptoms and the implantation is critical to the success rate. The same can be expected with regards to the stimulation of the gasserian ganglion (Kustermans et al. 2017).

## 6 CONCLUSION

These case reports show that stimulation of the gasserian ganglion is a successful, minimally invasive, and non‐destructive treatment in refractory trigeminal neuropathy and should be considered earlier in the treatment algorithm of trigeminal neuropathy.

## FUNDING INFORMATION

No funding was received for this work.

## CONFLICT OF INTEREST

The authors declare no conflicts of interest.

### TRANSPARENT PEER REVIEW

The peer review history for this article is available at https://publons.com/publon/10.1002/brb3.2379
